# The utilization of hydroxychloroquine to reduce the main signs and symptoms of COVID-19 patients, a cross-sectional study

**DOI:** 10.1016/j.amsu.2021.102867

**Published:** 2021-09-16

**Authors:** Salem Alsuwaidan, Ziad A. Memish, Faisal Alaklobi, Kholood Khan, Hamdan N. Alajami

**Affiliations:** a Research and Innovation Center, King Saud Medical City, Ministry of Health, Riyadh, Saudi Arabia; b College of Medicine, Alfaisal University, Riyadh, Saudi Arabia; c Medical Affairs, King Saud Medical City, Ministry of Health, Riyadh, Saudi Arabia; d Obstetrics and Gynecology, King Saud Medical City, Ministry of Health, Riyadh, Saudi Arabia; e Pharmaceutical Services Administration, King Saud Medical City, Ministry of Health, Riyadh, Saudi Arabia

**Keywords:** COVID-19, Hydroxychloroquine, Area under the curve (AUC), Fever, Cough, Shortness of breath

## Abstract

Hydroxychloroquine (HCQ) and chloroquine were found to have positive results in some non-randomized clinical trials with more benefit in decreasing the viral load of COVID-19. HCQ is a lysosomotropic and lipophilic drug that can penetrate cell membranes, and accumulates in the acidic lysosomes. The high concentration of alkaline HCQ increases the pH in lysosomes from the normal levels of 4.7–4.8 to 6 which leads to inhibition of lysosomes functions and thus, prevents the entry of coronavirus into cells.

**Objectives:**

The main aim of this study is to find out the appropriateness of using HCQ in asymptomatic/mildly symptomatic COVID-19 positive patients in an attempt to reduce the development of signs and symptoms of COVID-19 and severe disease.

**Methodology:**

Randomized selection, open-label trial to evaluate the efficacy of HCQ for patients presenting with asymptomatic COVID-19 upon diagnosis. Cases that met the inclusion criteria were divided into two arms [102 subjects to take HCQ (a loading dose of 400 mg twice daily given orally, followed by a maintenance dose of 200 mg twice daily for 4 days), and 100 subjects were used as a control group]. A follow-up for all the participants on daily basis for 14 days for any signs and symptoms (fever, cough, and shortness of breath). The main variables are action profile (represented by Area under the curve (AUC) for fever, cough, and shortness of breath statistically analyzed to differentiate between the two groups.

**Results:**

Data in this study showed that HCQ was effective in reducing body temperature from the first day to the fifth day; this positive effect was significant with (p < 0.001) compared with subjects who didn't receive HCQ. While there was no significant effect on cough or Shortness of breath.

**Conclusion:**

The recommendation of this study is to utilize HCQ to all subjects with asymptomatic COVID-19 infection providing that these subjects are within the inclusion criteria of this study. There was no adverse drug reaction observed for HCQ on daily follow-up.

## Highlights

• The debate for the use or not to use hydroxychloroquine for COVID-19 patients is still valid.
• A recommendation to utilize HCQ to all subjects with asymptomatic COVID-19 with fever.
• Utilization of HCQ should be within the inclusion criteria of this study.
• It had been noticed that there was no adverse drug reaction observed for HCQ on daily follow-up.

## 1 Introduction

Coronaviruses are a collection of RNA viruses usually associated with mild respiratory and gastrointestinal symptoms. Over the last 40 years, three strains emerged capable of inducing severe respiratory diseases. Severe acute respiratory syndrome coronavirus (SARS-CoV), Middle East respiratory syndrome coronavirus (MERS-CoV), and severe acute respiratory syndrome coronavirus-2 (SARS-CoV-2), causing coronavirus disease 2019 (COVID-19) [1]. COVID-19 was first identified in December 2019 in Wuhan, China, and has resulted in an ongoing pandemic. Globally, as of 31st Aug 2021, there had been 218 countries and territories around the world and 2 international conveyances and about 215 million confirmed cases of COVID-19, including 4.5 million deaths, reported to WHO. The standard method of diagnosis is by real-time reverse transcription-polymerase chain reaction (rRT-PCR) from a nasopharyngeal swab. Common symptoms include fever, cough, fatigue, shortness of breath, and loss of smell and taste. COVID-19 can spread rapidly and affects the upper and the lower respiratory tract as mostly but can affect many organs in the body because the virus accesses host cells via the enzyme angiotensin-converting enzyme 2 (ACE2) [2,3].

At present, the mainstay of treatment for COVID-19 thus far has been mainly supportive. Scientists conducted several clinical trials on repurposed old drugs to respond to this pandemic. At least two non-randomized studies that have used the combination of azithromycin and HCQ noted positive results in decreasing the viral load of COVID-19. Although HCQ received early EUA from the US FDA this authorization was later revoked due to lack of efficacy in RCT. Until today only Remdesivir and hydrocortisone have been approved by the Food and Drug Administration (US FDA) to be used as a treatment of choice to treat adolescent and adults hospitalized with COVID-19 [[4], [5], [6]]. However, in the long term, effective vaccines are needed to overcome the effects of the pandemic.

Hydroxychloroquine sulfate, is an analog of chloroquine, was first synthesized by introducing a hydroxyl group at the end of the side chain of chloroquine. HCQ is used for the treatment and prophylaxis of malaria as well as in the treatment of autoimmune diseases, such as rheumatoid arthritis and systemic lupus erythematosus [7]. The underlying mechanism of action of HCQ remain largely unknown, however, the previous studies have proposed that HCQ can inhibit the SARS-CoV through different steps. By increasing lysosomes pH may interfere with toll-like receptor signaling, and thus will reduce the production of cytokines and, in particular, pro-inflammatory cytokines; moreover, HCQ can alleviate the inflammatory response in COVID-19 patients [8]. HCQ is a lysosomotropic and lipophilic drug that is able to penetrate cell membranes, which accumulates in the acidic lysosomes. The high concentration of alkaline hydroxychloroquine increases the pH in lysosomes from the normal levels of 4.7–4.8 to 6 which leads to inhibition of lysosomes functions and thus, prevents the entry of coronavirus into cells [8,9]. In addition, HCQ can inhibit viral replication, decrease viral binding to target cells through impairing glycosylation of the ACE2 receptors and spike protein as well as inhibit virus assembly and release [10]. On the other hand, with a lower concentration of HCQ, it has the affinity to inhibit nucleic acid sensors, including cyclic GMP-AMP synthesis. This potential benefit of HCQ may cover its action as antiviral therapy [11].

HCQ is considered a safe drug and the toxicity is usually mild, infrequent, and usually reversible. The most common side effects are retinopathy, myopathy, and cardiotoxicity including the development of cardiomyopathy in patients with rheumatic diseases and rhythm disorders, as well as gastrointestinal effects including diarrhea, nausea, and vomiting. HCQ was demonstrated to be more potent and safer than chloroquine in inhibiting SARS-CoV-2 infected patients with “a loading dose of 400 mg twice daily given orally, followed by a maintenance dose of 200 mg twice daily for 4 days” [12,13].

The antiviral efficacy of HCQ was demonstrated after 5 days follow-up, where HCQ had a significantly faster time to body temperature recovery, cough remission and was associated with improvement in chest imaging findings [14]. HCQ showed high affinity as antiviral by inhibiting the growth of SARS-CoV-2 in vitro; these promising results had been confirmed with other anti-rheumatic medications such as colchicine as a potential therapeutic tool against COVID-19 which also has a high affinity for binding to the β-tubulin subunit and thus block microtubule polymerization, moreover, it has anti-inflammatory properties that may mitigate lung injury [15,16].

The purpose of this study is to decide about the appropriateness of utilizing HCQ for COVID-19 subjects at the time of diagnosis, defined as either:

• asymptomatic patients with positive PCR
• patients with sudden onset fever and/or acute respiratory symptoms (eg, cough, dyspnea) and have the intention to receive the COVID-19 test.
• those who met at least one of the following criteria:
  1. Having a history of travel to or residence to location reporting community transmission of COVID-19 disease; or
  2. Having contact with a confirmed or probable COVID-19 case including through work in health care settings.

In another simple definition, all subjects who have positive polymerase chain reaction (PCR) test but managed as an outpatient.

### 1.1 Objectives and Aim

The available clinical research on HCQ demonstrated some promising results in non-randomized controlled trials and potent activity by inhibiting SARS-CoV-2 infected patients and in patients with confirmed COVID-19. The aim of this study is to find out the appropriateness of utilizing HCQ for asymptomatic subjects with COVID-19, and the possibility to reduce the development of the main signs and symptoms of COVID-19.

## 2 Methodology

Simple randomization was applied on 202 subjects to either arm (102 subjects receiving HCQ and another 100 subjects were without HCQ neither any medications). Subjects visiting the diagnosis centers for COVID-19 were approached to participate before being COVID-19 positive to sign a consent form for their acceptance to enroll in the study. When the subjects were confirmed COVID-19 positive, HCQ was administered with a loading dose of 400 mg twice daily given orally, followed by a maintenance dose of 200 mg twice daily for four days, this is for the treatment group. Daily follow-up of patients on treatment to monitor fever (body temperature), shortness of breath, and cough for the first five days (starting from the day of testing), then a follow-up for all participants at days 7, 10, and 14.

### 2.1 Inclusion criteria

• Subjects with confirmed positive PCR test for COVID-19.
• Age above 12 years and older.
• Afebrile with no constitutional symptoms.
• Willing and able to comply with scheduled visits, treatment plan, and other study procedures.

### 2.2 Exclusion criteria

• Chronic disease (heart disease, DM) or taking any medications known to prolong the QT interval
• Pregnant or breastfeeding (avoid pregnancy while taking the study drug and for at least 30 days after the last dose of study drug)
• Allergies from 4-Aminoquinolines or known hypersensitivity to any component of the study drug
• Significant liver disease (defined as known cirrhosis or elevated transaminases of at least 3-fold upper limit of normal)
• Significant renal disease (defined as serum creatinine known to be > 2.0 mg/dl or on dialysis).
• Having a prior history of blood disorders such as aplastic anemia, agranulocytosis, leukopenia, or thrombocytopenia.
• Having a prior history of glucose-6-phosphate dehydrogenase (G-6-PD) deficiency.
• Having dermatitis, psoriasis, or porphyria.
• The patient currently being treated with efavirenz, nelfinavir, or fluconazole
• Or taking Digoxin, Mefloquine, methotrexate, cyclosporine, praziquantel, antacids and kaolin, cimetidine, ampicillin, Insulin or antidiabetic drugs, arrhythmogenic drugs, antiepileptic drugs, loop, thiazide, and related diuretics, laxatives and enemas, amphotericin B, high dose corticosteroids, and proton pump inhibitors, neostigmine, praziquantel, pyridostigmine, tamoxifen citrate.

### 2.3 Study location and subjects' recruitment

The study took place at the King Saud Medical City- Riyadh where the participants were recruited after signing the consent. Simple randomization was applied for the first 10 subjects in each arm, to find out the feasibility of this research, then no specific randomization was applied due to many withdrawn subjects. A total of 684 suspected subjects were enrolled in this study, but only 202 continued the follow-up and completed the duration of the study. Most of the withdrawals were for no specific reason, others were due to lack of eligibility or having negative PCR.

### 2.4 Sample size

Since HCQ is not indicated for suspected COVID-19 patients; this study was conducted on 10 subjects in each arm receiving and not receiving HCQ to find out the feasibility of this medication. After confirming the safety and efficacy of HCQ within the same inclusion and exclusion criteria; it has been decided then to increase the sample size from 10 subjects in each arm to 100 subjects in each arm.

### 2.5 Study procedure and data collection

Data were collected according to the response of the study participants in terms of the followings:

• Fever (body temperature) to use the exact body temperature by getting it from a thermometer. The subject or a representative receives the call used to inform the investigator about the average temperature per day (from days 1–5, 7, 10, and 14).
• The severity of cough (0–10): where zero if the subject has no cough, while 10 is used if the cough awakens the subject from sleep. Again, in between 0 and 10 will depend on the description of the subject (the repetitiveness of the cough and how violent air is released from the lungs) and the judgment of the investigator receiving the call to give a grade from 0 to 10.
• Shortness of breath (SOB) (0–10): Zero if the subject has no SOB and 10 if the subject needs mechanical ventilation. In between, 0–10 depends on the description of the subject (such as using a water evaporator to get a breath) and the judgment of the investigator receiving the call to give a grade from 0 to 10. This was applied to all subjects of the study in the two arms per day (from days 1–5, 7, 10, and 14).

Daily contact of the participants to follow their body temperature, measured by a thermometer, SOB (0–10), and cough (0–10); the subject used to express their feelings and to give a grade for SOB and cough after simple education by the investigators. A follow-up for all the 202 subjects for continuous 5 days, day 7, day 10, and day 14, where the data were collected in an excel sheet for analysis.

### 2.6 Statistical analysis

• Data entered daily after calling each individual subject in both arms. All data were registered in an excel sheet, then transferred to SPSS-26 for analysis. The following statistical analysis was applied:
• The area under the curve (AUC) for everyone regarding fever, SOB, and severity of cough, then compare between the two arms.
• Student t-test to compare between the means of the two arms. This will apply to fever profile, SOB, and severity of cough profiles.

### 2.7 Trendline and AUC calculation

**Fig. 1:**
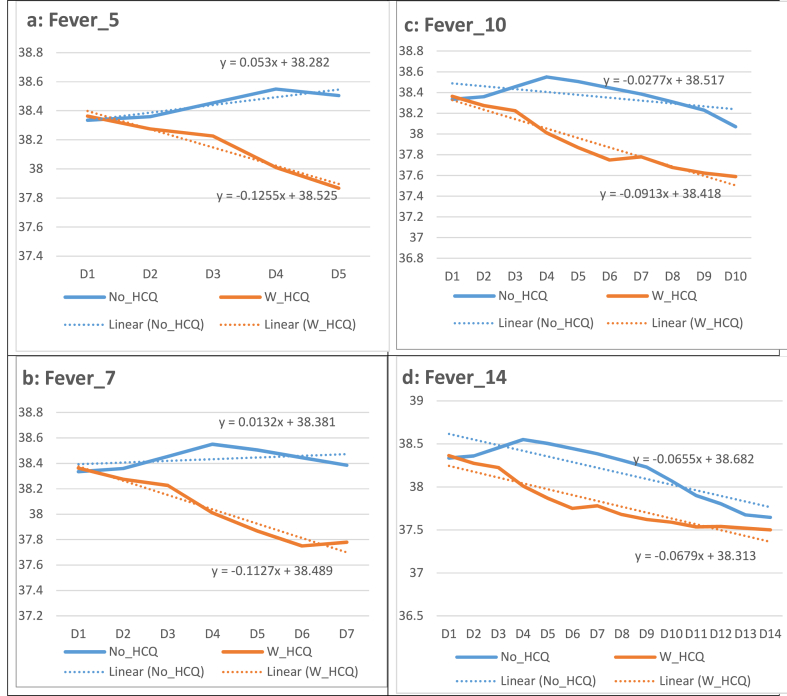
Action profile for **fever** indicating the mean results of 102 subjects receiving HCQ, compared with 100 subjects with no HCQ. The two profiles (subjects with and without HQC) represented the fever from day 1 on going to day 5 as in “a” showed the average action profile for fever for five days, it also showed the linear relationship for both profiles including the equation used as a factor to calculate the AUC. The same conclusion for “b”, “c” and “d” for 7 days, 10 days and 14 days consequently.

By using excel, create a best-fit straight trend line for the averages of fever, SOB, and severity of cough along 5 continuous days, seven, ten, and 14 days. Fig. 1 showed the trend line for each group (receiving HCQ and not receiving HCQ; for fever, SOB and cough); the equation of the straight line where using the slope as the factor to calculate the AUC for each subject.

## 3 Results

This study was conducted on 202 subjects with confirmed COVID-19 based on a positive PCR test. Their age was 48.46 years (±15.3 SD). The area under the curve (AUC) for each profile had been calculated representing the action profile for HCQ on fever “FAUC” for five days (1–5 days), then day 7, 10, and day 14 presented as FAUC5_1, FAUC7_1, FAUC10_1, FAUC14_1 consequently for subjects receiving HCQ and FAUC5_0, FAUC7_0, FAUC10_0, FAUC14_0 consequently for subjects not receiving HCQ. The area under the curve (AUC) also been calculated representing the action profile for HCQ on Shortness of breath “SAUC” and for cough “CAUC” with HCQ suffixed with"_1″ or with no HCQ "_0". There were 102 subjects receiving HCQ and 100 subjects represented the other group not receiving HCQ. The action profile of any variables representing the efficacy of HCQ indicated as AUC. Demographic data representing a number of participants including their ages and genders were shown in Table 1.

**Table 1:** Simple description for the demographic data representing number of participants receiving HCQ or not receiving HCO in each arm, their sex, average of age.

	Total n = 202		With HCQ n = 102		No HCQ n = 100	
	No.	Percentage	No.	Percentage	No.	Percentage
Male	126	62.4%	75	73.5%	51	51%
Female	76	37.6%	27	26.5%	49	49%
Age	48.5 ± (14.5 SD)		48.56 ± (13.7 SD)		48.46 ± (15.3 SD)	

Fever is considered as one of the main signs of viral infection; the average temperature for subjects on HCQ on the first day was 38.36 °C compared with those with no HCQ on the first day also was 38.34 °C. The average body temperatures in both groups were declined to 37.87 °C and 38.51 °C consequently on day 5. The declining action for the average temperature was represented in measuring the AUC for each individual, then the average of the AUC was calculated for day 5 ″FAUC5_1″ for subjects with HCQ and “FAUC5_0″ for subjects with no HCQ. There is no specific duration for cut-off to reduce the body temperature; however, fever started to decline to start from the first day within the group receiving HCQ then became very obvious with a significant difference (p < 0.001) on day 5 for the group receiving HCQ compared with the group not receiving HCQ. This significant difference in body temperature in comparing the mean of the group with HCQ and the group without HCQ became less different on day 7, then much less on day 10, and became with a minor difference “not significant” on day 14 according to the results shown in Table 2 and the profile actions are shown in Fig. 1. The most important part in Fig. 1 that can be achieved is the linear equation introduced by trendline to have the slope used as a factor to calculate the area under the curve (AUC) for each variable in terms of numerical value; then AUC represents the action profile of fever along 14 days follow-up. Results showed that there was no specific action of HCQ on the age categories or gender.

**Table 2:** Data representing the mean of Area Under the Curve for the action profiles for a group of subjects receiving hydroxychloroquine (HCQ) and another group not receiving HCQ; illustrating the mean data (for Fever AUC, Cough AUC, and Short of breath AUC) including standard error and standard deviation for the days 5, 7, 10 and day 14.

	With NO HCQ (n = 100)			With HCQ (n = 102)			
Day	**Mean**	St. Err	St. Dev	**Mean**	St. Err	St. Dev	P value
FAUC5	**−0.0225**	0.01	0.08	**−0.1553**	0.03	0.26	0
FAUC7	**0.0012**	0.00	0.04	**−0.2301**	0.03	0.33	0
FAUC10	**−0.0367**	0.01	0.13	**−0.5214**	0.17	1.73	0
FAUC14	**−0.3164**	0.04	0.41	**−0.4101**	0.04	0.42	0.4041
CAUC5	**−0.3008**	0.05	0.47	**−0.1096**	0.05	0.49	0.3306
CAUC7	**−0.5171**	0.07	0.69	**−0.2162**	0.07	0.74	0.2241
CAUC10	**−0.8871**	0.11	1.05	**−0.5174**	0.12	1.22	0.0647
CAUC14	**−0.7336**	0.08	0.80	**−1.0963**	0.20	2.05	0
SAUC5	**−0.2214**	0.05	0.47	**−0.2452**	0.05	0.54	0.059
SAUC7	**−0.3645**	0.06	0.64	**−0.3736**	0.09	0.87	0.0015
SAUC10	**−0.7337**	0.10	1.04	**−0.5095**	0.11	1.11	0.2572
SAUC14	**−1.3311**	0.15	1.49	**−0.9288**	0.17	1.74	0.0584

Note: The number suffixed each AUC representing of the day number for 5,7,10,and 14.

while FAUC = Fever AUC; CAUC= Cough AUC; and SAUC = Short of Breath AUC.

Data in this study showed that HCQ has a positive action by reducing body temperature from the first day along the five days with HCQ intake for the subject receiving HCQ; this positive action was with a very significant action made a difference with (p < 0.001) compared with subjects who didn't receive HCQ.

**Fig. 2:**
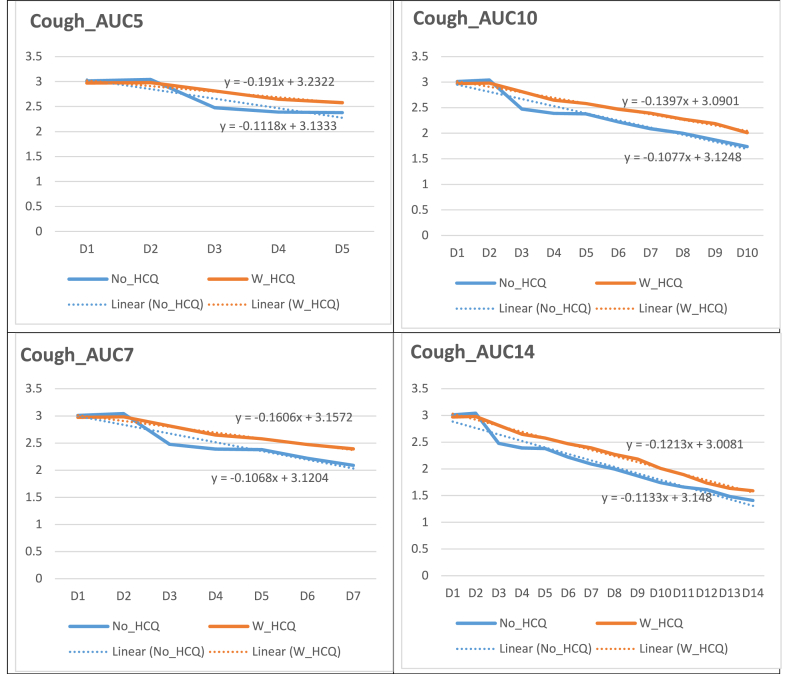
Action profile for **cough** indicating the mean results of 102 subjects receiving HCQ, compared with 100 subjects with no HCQ. The two profiles (subjects with and without HQC) represented the cough severity from day 1 on going to day 5 as in “a” showed the average action profile for fever for five days, it also showed the linear relationship for both profiles including the equation used as a factor to calculate the AUC. The same conclusion for “b”, “c” and “d” for 7 days, 10 days and 14 days consequently.

The severity of cough was also represented as a profile in subjects receiving HCQ and another group for the subjects not receiving HCQ. Subjects who participated (both arms) in this study used to express the severity of cough from zero up to ten; so that if the subjects used to wake up from sleeping, described the severity of cough as 10 and also depends on how frequent is the cough to make assessment out of 10 on the daily manner, decreasing to zero if there is no cough. Subjects receiving HCQ are not in favor of reducing their severity of cough –in fact – those who didn't receive HCQ showed better cough profile (less severity) compared with those who received HCQ in terms of all days 5, 7, 10, and 14. The action profile for the severity of cough (Fig. 2) showed a similarity of action during the first 10 days although the profile of subjects not receiving HCQ is better, where the reduction of severity of cough is less compared with those receiving HCQ but with no significant difference.

**Fig. 3:**
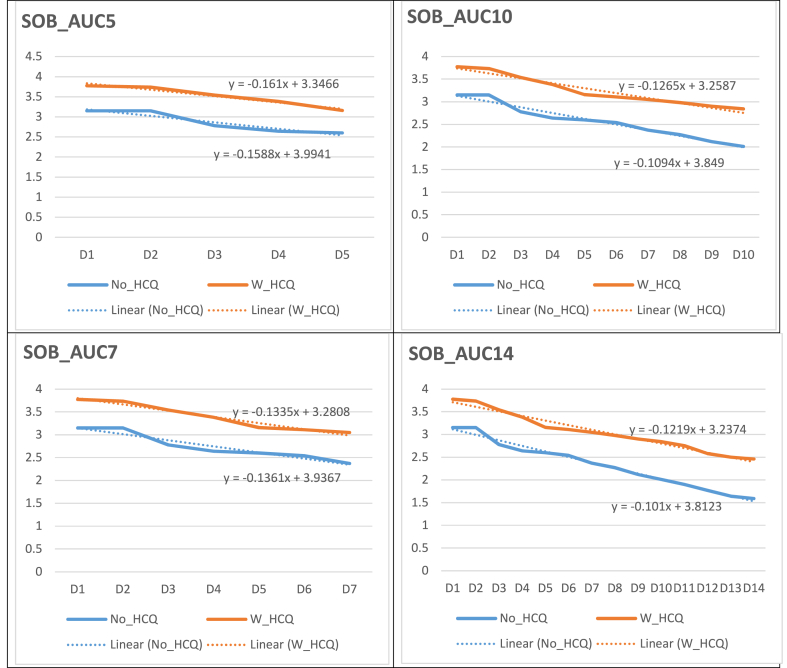
Action profile for the **Short of breath (SOB)** indicating mean results of 102 subjects receiving HCQ, compared with 100 subjects with no HCQ. The two profiles (subjects with and without HQC) represented the SOB from day 1 on going to day 5 as in “a” showed the average action profile for fever for five days, it also showed the linear relationship for both profiles including the equation used as a factor to calculate the AUC. The same conclusion for “b”, “c” and “d” for 7 days, 10 days and 14 days consequently.

Shortness of breath (SOB) was also represented as action profiles according to the patient's assessment, though that patients are the best person to evaluate themselves after the education of the investigator. The assessment for SOB was expressed as 10 if the subject being ventilated, decreasing to 6–8 if the subjects need to get evaporated water to facilitate their breathing process, down to occasionally SOB to get zero if there is no SOB. The results (Fig. 3) showed an almost parallel declining profile for SOB results, giving an indication that both groups have a similar AUC which means that HCQ has no favorable action towards reducing SOB.

Hydroxychloroquine has a negative impact (no influence) on sex (male or female), neither on age categories. Zero complain in relation with adverse drug reaction after hydroxychloroquine utilization in all subjects (n = 102).

## 4 Discussion

HCQ has less toxicity compared with other aminoquinoline and is used as anti-malaria and anti-inflammatory agent, for rheumatoid arthritis and lupus erythematosus. HCQ has shown a potential broad-spectrum antiviral activity [17,18] with one or more from the following mechanism of action:

• HCQ could interfere with the glycosylation of the angiotensin-converting enzyme 2 (ACE2) receptor, preventing the virus from binding to its target cells.
• pH-dependent mechanism of entry into target cells that can interfere with SARS-CoV-2 acidification of lysosomes.
• HCQ could inhibit the phosphorylation (activation) process which is required by viruses to achieve their replication cycles.
• HCQ has the affinity to increase the soluble viral antigens in the cytosol of dendritic cells and enhance a cytotoxic CD8^+^ T cell response against them.
• HCQ has the affinity to reduce the risk of thrombosis by inhibition of antiphospholipid antibody binding and reducing the procoagulatory state.

The absence of specific antiviral for COVID-19 gave the debatable medication “HCQ” a window to be used in a multi-center study that concluded that HQC alone was associated with a reduction in COVID-19 associated mortality [19].

On the other hand, it is not possible to state with confidence the precise efficacy and safety of HCQ in the treatment of COVID-19 at any time during the course of the disease. In addition to the uncertainty about time of administration, duration of treatment, prescribed dose, patients' condition, concomitant drug use, and others [20]. Another study showed that HCQ administration was not associated with a significantly lowered or an increased risk of the composite endpoint of intubation or death [21]. These results and others evaluating the effect of HCQ do not support its utilization in patients with COVID-19, due to major methodological weaknesses [22].

The results of this study proved that HCQ has a positive action towards decreasing body temperature in the subjects receiving HCQ in a significant manner (p < 0.0001) compared with the subjects not receiving HCQ. The action of HCQ by reducing fever started from the first HCQ dose received by subjects and being very significant on day 5 along the way to day 10 as action profile. This study proved that HCQ has a positive effect towards reducing the high body temperature profile to normal. The action of aminoquinolines in treatment of malaria fever [23], anginose glandular fever [24], and other causes of inflammatory fevers. Although another study illustrated that the HCQ have no therapeutic utilization in patients with COVID-19 infection [25], the recommendation of this study is to utilize HCQ for subjects with COVID-19 and having fever, provided that subject should receive HCQ within the same criteria of this study. Moreover, the ECG could be important after the first loading dose of HCQ 400 mg twice daily.

On the other hand, the action profile of HCQ on the severity of cough was also studied and showed that the action profile for subjects with or without receiving HCQ was the same. In fact, subjects not receiving HCQ illustrated better action profiles compared with subjects receiving HCQ, this change was not significantly different. There was only one case report that HCQ may induce dry cough in a patient with SLE receiving HCQ [26]. The last action profile was shortness of breath associated in subjects with COVID-19 that both arms were declining in a parallel profiles indicating both groups have similar action where the group receiving HCQ have no favorable action compared with those who did not receive HCQ.

This study is in favor to dispense HCQ to all subjects with COVID-19 providing these subjects should have similar inclusion criteria of this study, specifically if the subject with COVID-19 is with high body temperature.

## 5 Conclusion

Dispensing of HCQ to be with caution taking in consideration drug interaction, certain criteria for subjects receiving HCQ, electrocardiogram (ECG) is therefore recommended after starting the first dose. In other words, subject selection must be set for the utilization of HCQ to end up with high safety putting in mind that HCQ is not the drug of choice for COVID -19 but the utilization of HCQ was to reduce body temperature to normal. In addition, to put in mind that HCQ is ineffective towards cough or shortness of breath induced by COVID-19. It is also noticed that age and gender have no influence on the HCQ action profile. The recommendation of this research is to utilize HCQ to all subjects with suspicious COVID-19 providing that these subjects be within the inclusion criteria of this study. There was no experience of any known adverse drug reaction observed for HCQ on daily follow-up.

## Ethical consideration

Evidence of a personally signed and dated informed consent document indicating that the subject (or a legally acceptable representative) has been informed of all pertinent aspects of the study prior to initiation of any subject-mandated procedures. The IRB approval (H1RI-15-Apr20-02) had been modified when the investigators had positive achievement with the utilization of HCQ in the pilot study to increase the sample size from 10 subjects in each arm to 100 subjects in each arm.

## Sponsorship

King Abdulaziz for Science and Technology (KACST) has financially supported this research project.

## Conflicts of interest

This is to confirm that all authors have no conflict of interest, and they are all working full time job in the King Saud Medical City.

## Sources of funding

This research have been approved and supported financially by the King Abdulaziz for Science and Technology (KACST) as fast track research grant for COVID-19.

Sponsorship Number: GRANTS- MOH-00020-05-01-01.

Date: 17/6/2020.

## Ethical approval

Ethical approval was obtained:

Proposal Reference No.: H1RI-15-Apr20-02 ON Date: June 14, 2020; INSTITUTIONAL REVIEW BOARD (IRB) IRB Registration Number with KACST, KSA: H-01-R-053 IRB Registration Number U.S. Department of HHS IORG #: IORG0010374; and amendment for increasing number of subjects in each arm from 10 each arm to 100 in each arm as hydroxychloroquine is not indicated for COVID-19 subjects. Amendment was obtained after the approval of safety of HCQ for the selected subjects according to specific criteria.

## Consent

A written consent form was obtained from each participants in this research project including those who didn't receive hydroxychloroquine.

Authors confirmed that all procedures practiced in this research were according to good clinical practice standards.

## Author contribution

Please specify the contribution of each author to the paper, e.g. study concept or design, data collection, data analysis or interpretation, writing the paper, others, who have contributed in other ways should be listed as contributors.

**Table:** 

Author contribution	Salem	Ziad	Faisal	Khlood	Hamdan
writing research prposal	✓	✓	✓	✓	✓
Patient recruitment			✓	✓	✓
Patient consenting				✓	✓
Data collection			✓	✓	✓
Data analysis	✓	✓	✓		
Data interpretation	✓	✓	✓		
Writing the manuscript	✓	✓	✓		

## Registration of research studies

1. Name of the registry: researchregistry6926
2. Unique Identifying number or registration ID: 6926; Date June 27, 2021
3. Hyperlink to your specific registration (must be publicly accessible and will be checked): https://www.researchregistry.com/browse-the-registry#home/

## Guarantor

King Saud Medical City (KSMC)-Research and Innovation Center has full responsibility for conducting this study. KSMC is the largest hospital in Saudi Arabia, with 1500 beds of which 180 beds are in the intensive-care unit beds. It is also called Riyadh Central or Shumaisi Hospital it is located in Riyadh - Al Imam Abdul Aziz Ibn Muhammad Ibn Saud, Ulaishah, Riyadh, Alleesha District; Postal Code:12746, P.O.Box: Phone: 009664355555–0582181818 and the KSMC website is www.ksmn.med.sa. KSMC is a governmental sector under the umbrella of Ministry of Health considered as non-profit organization.

## Declaration of competing interest

The authors declare that there are no existing commercial or financial relationships that could, in any way, lead to a potential conflict of interest.

## References

[1] Dhama K., Sharun K., Tiwari R., Sircar S., Bhat S., Malik Y.S., Rodriguez-Morales A.J. (2020). ‏. Coronavirus disease 2019–COVID-19. Clin. Microbiol. Rev..

[2] Machhi J., Herskovitz J., Senan A.M., Dutta D., Nath B., Oleynikov M.D., Kline P. (2020). The natural history, pathobiology, and clinical manifestations of SARS-CoV-2 infections. J. Neuroimmune Pharmacol..

[3] Stawicki Stanislaw P., Jeanmonod Rebecca, Miller Andrew C., Paladino Lorenzo, Gaieski David F., Anna Q Yaffee, De Wulf Annelies (2020 Apr-Jun). The 2019–2020 novel coronavirus (severe acute respiratory syndrome coronavirus 2) pandemic: a joint American college of academic international medicine-world academic council of emergency medicine multidisciplinary COVID-19 working group consensus paper. J. Global Infect. Dis..

[4] Joshua Geleris, M.D., Yifei Sun, Ph.D., Jonathan Platt, Ph.D., Jason Zucker, M.D., Matthew Baldwin, M.D., George Hripcsak, M.D., Angelena Labella, M.D., Daniel K. Manson, M.D., Christine Kubin, PharmD., R. Graham Barr, M.D., Dr.P.H., Magdalena E. Sobieszczyk, M.D., M.P.H., and Neil W. Schluger, M.D. Observational study of hydroxychloroquine in hospitalized patients with covid-19. N. Engl. J. Med. published on May 7, 2020. 10.1056/NEJMoa2012410 PMC7224609 32379955

[5] (2020). Efficacy and safety of hydroxychloroquine and azithromycin for the treatment of hospitalized patients with moderate to severe COVID-19 - full text view - ClinicalTrials.gov [internet]. Clinicaltrials.gov.

[6] Coronavirus (COVID-19) Update (2020). https://www.fda.gov/news-events/press-announcements/coronavirus-covid-19-update-daily-roundup-march-30-2020.

[7] McChesney E. (1983). Animal toxicity and pharmacokinetics of hydroxychloroquine sulfate. Am. J. Med..

[8] Mindell J. (2012). Lysosomal acidification mechanisms. Annu. Rev. Physiol..

[9] Yao X., Ye F., Zhang M., Cui C., Huang B., Niu P. (2020). In vitro antiviral activity and projection of optimized dosing design of hydroxychloroquine for the treatment of severe acute respiratory syndrome coronavirus 2 (SARS-CoV-2). Clin. Infect. Dis..

[10] Offringa A., Montijn R., Singh S., Paul M., Pinto Y.M., Pinto-Sietsma S.J. (2020). The mechanistic overview of SARS-CoV-2 using angiotensin-converting enzyme 2 to enter the cell for replication: possible treatment options related to the renin–angiotensin system. European Heart Journal-Cardiovascular Pharmacotherapy.

[11] Chandler L.C., Yusuf I.H., McClements M.E., Barnard A.R., MacLaren R.E., Xue K. (2020). Immunomodulatory effects of hydroxychloroquine and chloroquine in viral infections and their potential application in retinal gene therapy. Int. J. Mol. Sci..

[12] Devaux C., Rolain J., Colson P., Raoult D. (2020). New insights on the antiviral effects of chloroquine against coronavirus: what to expect for COVID-19?. Int. J. Antimicrob. Agents.

[13] Smith E., Klein-Schwartz W. (2005). Are 1–2 dangerous? Chloroquine and hydroxychloroquine exposure in toddlers. J. Emerg. Med..

[14] Montealegre-Gómez G., Garavito E., Gómez-López A., Rojas-Villarraga A., Parra-Medina R. (2020). Colchicine: a potential therapeutic tool against COVID-19. Experience of 5 patients. Reumatol. Clínica.

[15] Chams N., Chams S., Badran R., Shams A., Araji A., Raad M., Hajj Hussein I. (2020). COVID-19: a multidisciplinary review. Frontiers in public health.

[16] Bakadia B.M., He F., Souho T., Lamboni L., Ullah M.W., Boni B.O., Yang G., ‏ (2020). Prevention and treatment of COVID-19: focus on interferons, chloroquine/hydroxychloroquine, azithromycin, and vaccine. Biomed. Pharmacother..

[17] Pal A., Pawar A., Goswami K., Sharma P., Prasad R. (2020). Hydroxychloroquine and covid-19: a cellular and molecular biology based update. Indian J. Clin. Biochem..

[18] Arshad S., Kilgore P., Chaudhry Z.S., Jacobsen G., Wang D.D., Huitsing K., O'Neill W. (2020). Treatment with hydroxychloroquine, azithromycin, and combination in patients hospitalized with COVID-19. Int. J. Infect. Dis..

[19] Boralli V.B., da Silva R.G.M. (2020). COVID-19 pandemic-A narrative review of the potential roles of chloroquine and hydroxychloroquine. Pain Physician.

[20] Geleris J., Sun Y., Platt J., Zucker J., Baldwin M., Hripcsak G., Sobieszczyk M.E. (2020). Observational study of hydroxychloroquine in hospitalized patients with Covid-19. N. Engl. J. Med..

[21] Roustit M., Guilhaumou R., Molimard M., Drici M., Laporte S., Montastruc J.L. (2020). Chloroquine and hydroxychloroquine in the management of COVID-19: much kerfuffle but little evidence. Therapie.

[22] Adebayo R.A., Sofowora G.G., Onayemi O., Udoh S.J., Ajayi A.A. (1997). Chloroquine‐induced pruritus in malaria fever: contribution of malaria parasitaemia and the effects of prednisolone, niacin, and their combination, compared with antihistamine. Br. J. Clin. Pharmacol..

[23] Alexander J.G., Alexander P.J. (1975). Chloroquine for anginose glandular fever. J. Roy. Coll. Gen. Pract..

[24] Das L.K., Jambulingam P., Sadanandane C. (2008). Impact of community-based presumptive chloroquine treatment of fever cases on malaria morbidity and mortality in a tribal area in Orissa State, India. Malar. J..

[25] Mitja O., Corbacho-Monné M., Ubals M., Alemany A., Suner C., Tebe C., Admella P. (2020). A cluster-randomized trial of hydroxychloroquine for prevention of covid-19. N. Engl. J. Med..

[26] Verghese E., Haque U., Jabri A., Modugula S., Tewari S. (2020). A dry cough conundrum: a rare case OF pneumocystis jirovecii pneumonia IN systemic lupus erythematous patient ON hydroxychloroquine. Chest.

